# Surgery Without Scalpel: Histotripsy as a Non-Invasive and Non-Thermal Modality for Liver Tumor Ablation

**DOI:** 10.3390/jcm14186391

**Published:** 2025-09-10

**Authors:** Daniel Paramythiotis, Dimitrios Tsavdaris, Georgios Tsavdaris, Adam Hatzidakis, Kyriakos Psarras, Alexandros Mekras, Christos Georgiades, Antonios Michalopoulos

**Affiliations:** 1 First Propaedeutic Surgery Department, University General Hospital of Thessaloniki AHEPA, Aristotle University of Thessaloniki, 54636 Thessaloniki, Greece; tsavdaris@auth.gr (D.T.); amichal@auth.gr (A.M.); 2 Surgery Department, General Hospital of Thessaloniki Agios Pavlos, 55134 Thessaloniki, Greece; 3 Department of Radiology, University General Hospital of Thessaloniki AHEPA, Aristotle University of Thessaloniki, 54636 Thessaloniki, Greece; adamhatz@auth.gr; 4 Third Department of Surgery, University General Hospital of Thessaloniki AHEPA, Aristotle University of Thessaloniki, 54636 Thessaloniki, Greece; psarrask@auth.gr; 5 Department of General and Visceral Surgery, SHG-Klinikum Merzig, Academic Hospital of University of Saarland, 66663 Merzig, Germany; almekras@yahoo.gr; 6 Vascular & Interventional Radiology, Johns Hopkins University, Baltimore, MD 21218, USA; g_christos@hotmail.com

**Keywords:** histotripsy, liver, cancer, hepatocellular carcinoma, management, ultrasound

## Abstract

Liver malignancies are among the most prevalent cancers worldwide and can be managed using various therapeutic approaches. However, the available options for treating these malignancies are characterized by several limitations. Histotripsy, which was recently approved by the Food and Drug Administration (FDA), seems to be promising for overcoming these limitations. It is an emerging non-invasive, non-thermal ultrasound technology, which is based on the controllable initiation of cavitation from endogenous nanometer-scale gas pockets within tissues. Numerous preclinical studies as well as three clinical studies highlight this technique as feasible, safe, and effective. Among its advantages are the lack of thermal injury, its non-invasive nature, its immunopreserving and possibly immunostimulating ability, as well as the low number of complications that accompany it. Nonetheless, long-term clinical outcomes are still lacking, and further studies are needed to establish its definitive role in liver cancer treatment. In conclusion, histotripsy shows strong potential to become a transformative tool in liver oncology, but continued clinical evaluation is essential to validate its long-term efficacy and integration into standard care.

## 1. Introduction

Primary liver cancer remains a major global health challenge. In 2020, an estimated 905,700 new cases of liver cancer were diagnosed worldwide, and 830,200 deaths occurred, making it the sixth most common cancer and the fourth leading cause of cancer-related mortality globally [1]. Hepatocellular carcinoma (HCC) accounts for approximately 90% of these cases [2].

HCC originates mostly in cirrhotic livers with a background of hepatitis B virus (HBV) and hepatitis C virus (HCV), as well as non-alcoholic steatohepatitis (NASH) and alcohol-related liver disease (ALD), among others. Furthermore, it is a malignancy with a high metastatic potential and a tendency to be diagnosed at late stages, complicating its treatment and being associated with high mortality [3,4].

These malignancies can be treated with a variety of techniques. In early stage, surgical resection is the treatment of choice. This option is often accompanied by an impact on liver function, as well as carries significant perioperative morbidity and is not feasible in cirrhotic livers or metastatic disease [5,6,7]. Other treatment options include thermal ablation (e.g., radiofrequency and microwave ablation). However, these options are insufficient to treat many patients, with their success depending on factors such as tumor size, location, multifocality, or coexisting liver dysfunction [5,8,9]. Also notable are the complications of these procedures, such as thermal methods, which suffer from heat-sink effects adjacent to large vessels and can damage surrounding healthy parenchyma [8]. Stereotactic body radiation is another option in advanced disease but carries the risk of radiation-induced liver disease [8]. Other methods include transarterial therapy, which is characterized by limited efficacy in metastasis, as well as chemotherapy, to which tumors often appear resistant [7,10]. Immunotherapy appears to be more effective when used as a combination therapy, but this increases the toxicity.

In addition to primary liver malignancies such as HCC, the liver is a frequent site for metastases. These arise when malignant cells spread from a primary cancer elsewhere in the body, most commonly from colorectal, pancreatic, breast, and lung cancers, as well as melanoma, via the liver’s dual blood supply and unique microenvironment [11,12,13]. Colorectal cancer is the predominant source, although virtually any solid tumor can metastasize to the liver, and most metastatic lesions are adenocarcinomas [13]. Liver metastases are biologically and clinically distinct from HCC, with management strategies determined by the nature of the primary tumor, extent of hepatic involvement, and patient condition. For instance, resectable colorectal liver metastases are typically treated with surgical resection, often in combination with systemic chemotherapy and, in selected cases, targeted or immunotherapeutic agents. Accurate imaging plays a critical role in the diagnosis and staging of liver metastases, as therapeutic decisions hinge on the primary cancer type and surgical resectability, which differ substantially from the approach to HCC [14].

Histotripsy is a novel Food and Drug Administration (FDA)-approved non-invasive, non-thermal ablative method that employs ultrasound. High-power, focused acoustic waves are phased to cause tissue cavitation and subsequent mechanical disruption of the tissue transforming it into an acellular debris without reliance on thermal injury. By generating controlled microbubble clouds at the focal zone, histotripsy achieves submillimeter precision and sharp lesion boundaries, sparing connective structures such as bile ducts and blood vessels [15].

In this review, we will outline the biophysical principles and device configurations underlying histotripsy, summarize preclinical and clinical evidence of its efficacy and safety in hepatic tumors, compare its advantages and limitations relative to conventional therapies, and discuss future directions for integrating histotripsy into multidisciplinary liver cancer management.

## 2. Histotripsy: Principles and Mechanism of Action

Histotripsy is a purely mechanical, non-thermal tissue ablation technique that uses focused ultrasound to generate and control cavitation in targeted tissues. Unlike radiofrequency or microwave ablation, founded on resistive or dielectric heating, and thermal high-intensity focused ultrasound (HIFU), histotripsy applies short (<10 µs), high-pressure (>20 MPa) acoustic pulses with low duty cycles (≤1%) to seed and sustain cavitation bubble clouds with negligible heat deposition. Since the duty cycle is maintained well below 1%, tissue temperatures never rise toward the values needed for coagulative necrosis, and thus bioeffects are controlled by mechanical stresses rather than by thermal injury [16,17,18,19,20,21].

### 2.1. Acoustic Cavitation and Bubble Cloud Formation

The key to histotripsy is the controlled production of cavitation from endogenous nanometer-scale gas bubbles within tissues. Two mechanisms may be employed to create a dense cloud of bubbles within the focal region:

Intrinsic-threshold histotripsy: A single-cycle pulse with a peak negative pressure greater than the tissue’s intrinsic cavitation threshold (≈26–30 MPa for water-rich tissues) nucleates an instantaneous cloud of bubbles. Each bubble grows and then implodes violently to mechanically fractionate tissue [18,19,22,23,24,25].

Shock-scattering histotripsy: For negative peak pressures less than the intrinsic threshold (≈15–20 MPa), a probabilistically created statistical microbubble acts as a scatterer of incoming nonlinear shock fronts. The shock front high-frequency components invert and form local negative peaks above the cavitation threshold and backscatter to the transducer a fan-shaped cloud [18,19,22,23,24,25].

Bubble inflation occurs on a time scale of hundreds of microseconds, with diameters increasing from a few nanometers to over 100 µm, prior to subsequent rapid cavitation collapse. Such rapid volumetric oscillations impart extremely high local strain rates (>10^6^ s^−1^) and stresses (>1 MPa) upon neighboring cells, shearing cell membranes and the extracellular matrix with subcellular precision [18,19,22,23,24,25].

### 2.2. Device and Pulse Parameters

Histotripsy systems generally utilize transducers with low frequencies (0.5–3 MHz) to achieve a trade-off between penetration depth and focal resolution. Pulses are applied as bursts of 1–20 cycles, with repetition frequencies for the pulses between 1 Hz and 1 kHz, and total treatment dose adjusted by the number of pulses per focal location. Low duty cycle (<1%) is necessary to avoid thermal build-up, and real-time imaging (ultrasound or magnetic resonance imaging-MRI) coupled with the transducer assembly permits visualization of cloud bubble generation and precise targeting [16,17,18].

### 2.3. Mechanical Tissue Fractionation and Disintegration

**Figure 1:**
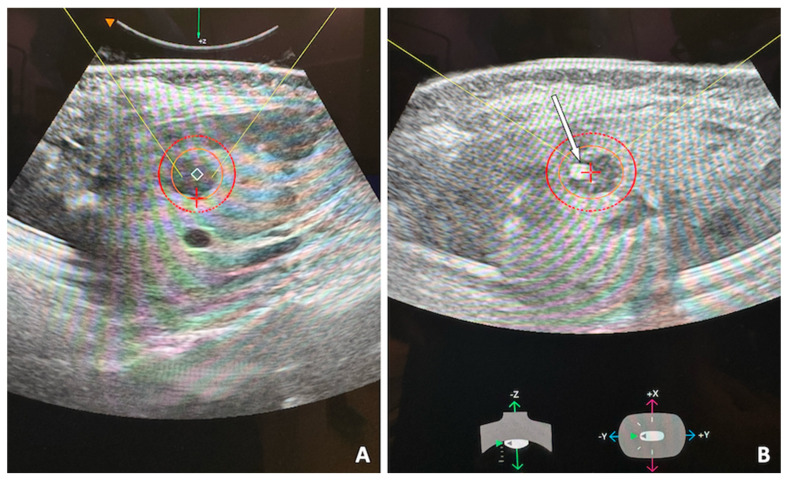
53-year-old female with single hepatic metastasis from breast cancer, refractory to systemic chemotherapy and referred for histotripsy. Planning ultrasound of the left hepatic lobe (**A**) shows the hypoechoic lesion. The inner orange circle denotes the selected ablation zone and the outer red circle adds a 5 mm ablation margin. Intra-procedural ultrasound image (**B**) shows the bubble cloud (white arrow) targeting the lesion. A robotic arm guides the ultrasound head to target first the distal portion of the mass and proceed superficially in a spiral trajectory, eventually covering the entire planned ablation zone.

**Figure 2:**
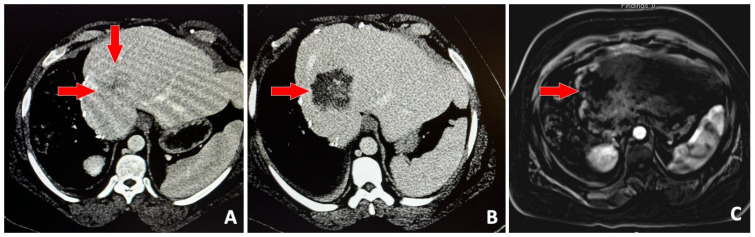
39-year-old male with history of right hepatectomy for metastatic colon cancer, presents with single hepatic metastasis and referred for histotripsy. Baseline contrast-enhanced CT (**A**) shows the 3.5 cm solid lesion (red arrows). Contrast-enhanced CT on post-procedure day #1 (**B**) shows no enhancement in the region that was targeted with histotripsy. Subtracted, contrast-enhanced T1-weighted MRI (**C**) 1-month post-procedure shows no evidence of viable tumor (red arrow).

As the cavitation bubbles expand and then implode, high-pressure shear fields and shear forces mechanically degrade targeted tissue into an acellular, viscous debris (Figure 1). Histotripsy lesions have distinct borders, even in the proximity of large vessels and ducts, as connective structures are more resistant to cavitation compared to cells. During the subsequent weeks, liquefied debris is phagocytosed by macrophages with minimal fibrosis or scar [16,17,21]. Follow-up imaging can reveal the real extent of necrosis (Figure 2).

### 2.4. Biological and Immunological Implications

Aside from mechanical ablation, histotripsy also causes release of damage-associated molecular patterns (DAMPs) high-mobility group box 1 (HMGB1), adenosine triphosphate (ATP), and heat-shock proteins that have the potential to activate and attract innate immune cells within the tumor microenvironment. Preclinical models demonstrate that these immunogenic signals can complement checkpoint inhibitors to generate systemic antitumor responses, which suggests a dual role for histotripsy of local control and immunomodulation [26]. It should be emphasized that unlike microwave ablation in which the high temperatures denature tumor-associated proteins, with histotripsy such tumor proteins remain mostly intact.

## 3. Preclinical and Clinical Evidence in Liver Oncology

### 3.1. Preclinical Evidence in Liver Oncology

Numerous preclinical studies have demonstrated that histotripsy, being a non-thermal, mechanical, focused ultrasound ablation technique, can offer accurate, non-invasive liver tumor ablation of well-defined lesions in liver tumors with sparing of critical structures and satisfactory safety and efficacy profiles [27]. Worlikar et al., one of the first histotripsy tumor survival rodent trials, displayed tumor burden reduction effects and increased survival compared to controls [28]. Qu et al. also demonstrated that histotripsy of liver malignancies can promote both local and systemic anti-tumor immune responses and enhance the effectiveness of checkpoint inhibitor immunotherapy [29]. Hendricks-Wenger et al. also demonstrated that increased doses of histotripsy led to more clearly defined treatment margins as well as enhanced tumor progression-free survival and overall survival compared to control groups [30,31]. Compared with conventional ablation therapies such as radiofrequency ablation (RFA) and microwave ablation (MWA), preclinical studies have shown that histotripsy produces more spherical ablation zones, has fewer biliary complications, and greater regression of the ablation zone over time, with at least equivalent procedural tolerance and cellular destruction to MWA [32,33].

### 3.2. Clinical Evidence in Liver Oncology

Three prospective clinical studies have been conducted on the use of histotripsy for liver tumor patients, including the first-in-human THERESA trial, the multicenter phase I/II HOPE4LIVER trial, and Wehrle et al. involving 230 patients across 9 centers, as summarized in Table 1 [34,35,36]. These trials have established the technical feasibility, safety, and initial efficacy of histotripsy in primary and metastatic liver malignancies, but long-term oncologic outcomes are still under investigation.

The results of the THERESA trial confirm that histotripsy is effective, technically viable, and demonstrates early efficacy for the treatment of liver tumors. In this first-in-human, multicenter phase I study, all enrolled patients (*n* = 8) with unresectable, multifocal primary or secondary liver tumors achieved the primary endpoint of technical success. The latter was defined as the creation of a zone of tissue destruction matching the planned ablation volume as assessed by MRI one day post-procedure. No device-related adverse events were observed up to the two-month follow-up indicating a good safety profile. In addition, sustained reductions in tumor markers were observed in two patients at eight-month follow-up, demonstrating early biological efficacy. The safety profile of histotripsy in the THERESA trial is thus favorable compared to the reported major complication rates of RFA and MWA, which stand at 2–7% [34].

The multicenter phase I/II HOPE4LIVER trial demonstrated a histotripsy technical success rate of 95% (42/44 tumors) and an index procedure–related major complications rate of 7% (3/44 patients) within 30 days, both meeting prespecified performance goals for safety and efficacy in the ablation of primary and metastatic liver tumors. The research included patients with a maximum of three tumors (≤3 cm in diameter), both HCC and metastases, who had multifocal disease and were not candidates for or had declined conventional treatments. The zones of ablation were well demarcated, and the mean of post-treatment zone diameter was 3.6 cm, showing effective treatment of the intended lesions [35].

At 1-year follow-up, local tumor control rates were 63.4% by primary assessment and 90% by post hoc imaging review, with overall survival at 1 year of 73.3% for HCC and 48.6% for metastatic disease, outcomes that are consistent with those reported for established locoregional therapies in similar patient populations. The safety profile was favorable, with most adverse events being minor and only six off-target damage events within 30 days, none resulting in mortality.

Wehrle et al. [36] evaluated histotripsy for liver tumor ablation in 295 patients across 18 centers and found a low overall rate of complications of 5.2% (12/230 patients), with most complications being minor (Clavien–Dindo grade ≤ II), and a rate of major complications of 1.3% (3/230 patients). All reported deaths occurred due to disease progression in patients treated with palliative intent and were not related to the procedure. Median and mean Comprehensive Complication Index (CCI) were both 0.00. All eight segments of the liver and the most diverse range of tumor types (colorectal metastases, neuroendocrine tumors, HCC, pancreatic, and breast metastases) were treated, with technical feasibility and generalizability. These results confirm histotripsy to be well tolerated and have a superb safety profile in the clinical setting, with extremely rare severe adverse effects and zero device-related deaths [36].

Long-term oncologic outcomes such as overall survival, disease-free survival, and local recurrence rates for histotripsy remain undefined in humans, as follow-up is limited and large-scale randomized comparative trials are lacking. In contrast, RFA and MWA have established long-term data, with 5-year overall survival rates for HCC and colorectal liver metastases ranging from 23 to 39% and local recurrence rates of 5–18% in large series [8,37].

Based on current preclinical and early clinical evidence, histotripsy is most beneficial in patient populations with liver tumors that are adjacent to major blood vessels or bile ducts, or in those at higher risk for thermal injury or heat sink effects from conventional ablation therapies. Histotripsy’s non-thermal, mechanical mechanism allows for precise ablation with preservation of critical structures, reducing the risk of biliary complications and collateral damage compared to RFA and MWA. Additionally, histotripsy has shown efficacy in ablating dense, fibrotic tumors such as cholangiocarcinoma, although higher treatment doses may be required.

### 3.3. Contraindications for Using Histotripsy

Histotripsy shares most of the contraindications observed with RFA and MWA, as its safe and effective application is influenced by similar technical, anatomical, and patient-related factors. One of the strongest limitations is the failure to delineate or target the tumor precisely with the use of ultrasound guidance. Since histotripsy relies on real-time imaging for precise delivery of focused ultrasound pulses, those patients with suboptimal acoustic windows, due to deeply located tumors, overlying bowel gas, or unfavorable body habitus, may be potentially less than optimal candidates. Inadequate tumor visualization may compromise both the safety and efficacy of the procedure [15,16,35,38]. Nevertheless, with strategic planning and optimal use of acoustic windows, it is possible to target such lesions safely and effectively, as shown by Wehrle et al. [36] who had successfully targeted all liver segments [36].

Another contraindication includes uncorrectable coagulopathy or major thrombocytopenia. Although histotripsy is safer than thermal ablation in anticoagulated patients since it is a mechanical, non-thermal procedure, it still carries an inherent risk of bleeding associated with any tissue-disrupting technique. Therefore, these patients with severe bleeding disorder should be carefully assessed before subjecting them to the procedure, and if a procedure is required, appropriate pre-procedure management is essential, particularly in cases of uncorrectable coagulopathy or major thrombocytopenia [15,32,39].

Histotripsy is not well suited for diffuse infiltrative diseases or cases with extensive tumor burden. As a focal treatment, it is currently best utilized in discrete, well-circumscribed lesions. Patients with extensive hepatic disease are unlikely to benefit from a procedure targeting one or even a few lesions. Patients with decompensated liver disease, and particularly those who are Child–Pugh class C, risk having worsening hepatic decompensation following any form of local ablative treatment, including histotripsy [15,16,35,38].

Anatomical proximity to critical structures also plays a role in contraindication of histotripsy in some cases. While histotripsy is less risky than thermal ablation for damaging important vessels or bile ducts, because of the cavitation mechanism, it remains risky in cases where tumors are near structures that could be damaged, such as the gallbladder or the gastrointestinal tract. In these situations, collateral damage could be disastrous [15,16,35,38]. The contraindications and limitations of histotripsy are summarized in Table 2.

### 3.4. Advantages over Traditional Therapies

Histotripsy represents a paradigm shift in focal liver tumor therapy by replacing thermal ablative and surgical excision options with a purely mechanical and non-invasive approach. Traditional resection and ablation techniques, whether open or percutaneous, depend on tissue excision or sustained thermal energy to induce coagulative necrosis. These methods inevitably damage non-target liver parenchyma, carry risks of bleeding, bile leakage, and heat-sink effects near large vessels, and often require significant recovery time [16,40]. Furthermore, RFA can be dangerous in patients with implanted electronic equipment like pacemakers because of possible electromagnetic interference, a problem not faced with histotripsy [8,41,42]. In contrast, histotripsy delivers microsecond-long, high-amplitude ultrasound pulses from outside the body, generating a controlled cavitation cloud at the focal point that liquefies tumor cells, transforming them into acellular debris. Because tissue temperatures remain near physiologic levels, there is no collateral thermal injury. Blood vessels and bile ducts are preserved due to their higher resistance to mechanical stress, and the risks associated with cavitation-induced pressure surges, charring, or thermal spread are effectively minimized [16,40]. Furthermore, histotripsy’s completely non-invasive approach eliminates the need for surgical incisions, thereby minimizing the risk of bacterial contamination, virtually eliminating intraoperative bleeding, and significantly reducing postoperative pain.

Moreover, histotripsy’s mechanism is inherently non-immunosuppressive and potentially immune-stimulatory, a property also observed with RFA and MWA. Mechanical fractionation releases intact tumor antigens and DAMPs into the local microenvironment without denaturing them by heat. Preclinical studies have demonstrated that this antigenic debris can recruit dendritic cells and CD8^+^ T-cells, leading not only to robust clearance of the treated lesion but also to systemic “abscopal” effects against distant metastases. By preserving the structural integrity of connective tissues, histotripsy facilitates rapid infiltration of immune cells and supports favorable remodeling rather than fibrotic scarring. This contrasts sharply with thermal ablation, which can denature proteins, impede antigen presentation, and leave a fibrotic rim that hinders immune cell access [43,44,45,46]. Clinically, a case report has also presented an abscopal response in a histotripsy-treated patient with liver tumors, demonstrating regression of untreated lesions and sustained reductions in tumor markers, confirming the potential for systemic immune benefit [47].

Finally, the practical implications of histotripsy for patient care are substantial. The absence of incisions or percutaneous probes allows treatments to be performed under light sedation or even local anesthesia in an outpatient setting, shortening hospital stays and accelerating return to normal activities. Although histotripsy in principle can be performed under local anesthesia or sedation, general anesthesia is in fact employed in the majority of centers since it guarantees that the patient remains completely still, which is of crucial importance for correct targeting. Because its therapeutic effect does not rely on cumulative heat exposure, histotripsy can be repeated safely, either in the same session to cover larger or multifocal tumors, or in subsequent sessions to treat recurrences, without adding appreciable risk to the patient. Real-time ultrasound guidance and the image fusion capabilities of histotripsy enhance procedural precision by allowing clinicians to visualize cavitation clouds and dynamically adjust the focus or pulse parameters during treatment. Taken together, these attributes position histotripsy not simply as an alternative to surgery or thermal ablation, but as a versatile, tissue-sparing modality with the potential to improve both oncologic outcomes and patient quality of life.

### 3.5. Recommended Selection Criteria for Histotripsy in Liver Tumor Ablation

Clinical application of histotripsy for liver tumor ablation requires careful patient selection for maximizing safety, efficacy, and therapeutic benefit. Based on clinical trial data and early real-world experience, several key criteria have been suggested.

- Maximum Size and Number of Liver Tumors

Histotripsy has been most effective in patients with tumors up to 5 cm in diameter, with both efficacy and safety most reliably optimal in this cohort. Tumors larger than 5 cm have also been treated on a case-by-case basis, although this is less common and needs to be carefully considered. The number of tumors treated in any one session is usually one to three, with most procedures treating one or two significant lesions [34,35].

ii. Child–Pugh Grading for Eligibility

Eligibility is generally restricted to patients with preserved liver function. Those with Child–Pugh class A or B cirrhosis are suitable candidates, but those with Child–Pugh class C are generally excluded due to the extremely high risk of hepatic decompensation. This eligibility criterion is comparable to the selection criterion for other liver-directed ablative therapies [34,35].

iii. Tumor Location Within the Liver

One of the major advantages of histotripsy is that it is a non-thermal mechanism and thus enables treatment of tumors in any hepatic segment, including those in very close proximity to large vessels, bile ducts, or the liver capsule. This enables it to overcome limitations such as the heat-sink effect of thermal modalities [30,35,48].

iv. Patient and Tumor Characteristics Modulating Immunomodulatory and Abscopal Effects

Patients with low metastatic burden, accessible lesions, and less fibrotic and antigenic tumors are most likely to achieve benefits from histotripsy for liver cancer and are immunocompetent [45,48]. Here, in addition to precise local ablation, histotripsy facilitates systemic immune activation with CD8^+^ T cell infiltration and abscopal effects. Tumors with well-defined antigens (e.g., AFP, HER2) and minimal stromal fibrosis, such as hepatocellular carcinoma or colorectal metastases, are most likely to respond. Conversely, patients with extensive disease, highly fibrotic tumors, or severe immunosuppression will not be likely to benefit systemically. Overall, careful selection of immunocompetent patients with accessible, antigenic, and biologically favorable tumors maximizes both local efficacy and immunomodulatory potential of histotripsy [45,48].

## 4. Challenges and Limitations

Despite its promising advantages, histotripsy faces limitations and technical and clinical hurdles that must be carefully addressed before it can be widely accepted as a mainstream therapeutic tool in liver oncology.

One of the significant technical hurdles is the accurate and effective delivery of acoustic energy into deeply seated liver lesions, especially those underneath rib shadows or above bowel gas. High-intensity ultrasound pulses required for cavitation are highly susceptible to acoustic aberration and attenuation in traveling through bone or air-filled tissues. Transcostal delivery, in particular, may result in defocusing and off-target cavitation at the expense of treatment effectiveness or inducing unintended harm to adjacent tissues. Animal model investigations have demonstrated that cavitation thresholds are not always consistently achieved in these anatomically complex regions, and ill-advised interactions with the diaphragm or lung may induce temporary adverse effects such as pulmonary hemorrhage [49,50,51]. Similarly, in the obese patient, supra-abdominal excess subcutaneous adipose tissue further attenuates the ultrasound beam, possibly limiting treatable depth or necessitating higher energy output, which presents additional safety considerations [52,53].

Another limitation is the variation in tissue susceptibility to cavitation. While histotripsy selectively ablates soft, parenchymal tissue, variations in liver composition among patients, e.g., cirrhotic fibrosis or tumor calcification, can affect cavitation dynamics. This variation can lead to non-homogeneous ablation zones and increase the risk of residual tumors. Therefore, treatment planning and dose modulation must be paid attention to and customized [17,31]. Furthermore, the impact of tumor microenvironment characteristics, such as vascular density, stromal composition, or immune cell invasion, on histotripsy effects, especially on heterogeneous or necrotic tumors, remains poorly understood [16,27,54].

Another practical limitation is that histotripsy procedures can require considerably more time to complete, compared with other available local ablative options such as RFA or MWA. The need for precise, focal targeting and gradual lesion coverage prolongs treatment duration, especially for larger or multiple lesions. This extended procedural time may increase patient discomfort, and limit throughput in busy clinical settings. It also has implications for resource utilization, scheduling, and overall healthcare costs, potentially impacting its adoption in centers with high patient volumes.

Biologically, although histotripsy appears to spare large structures and minimize off-target damage to adjacent parenchyma, concerns still linger regarding potential off-target damage. Theoretically, mechanical ablation of tumor tissue could redistribute viable malignant cells into the peritoneal cavity for superficial lesions, though no such event has been reported in early human trials [36]. Endothelial damage caused by cavitation is also bound to trigger localized thrombosis, especially in the vicinity of large hepatic vessels [36]. While these risks are minimal in preclinical trials, long-term follow-up data for recurrence rates, systemic toxicity, and disease-free survival are not yet available.

From a logistical perspective, histotripsy systems are currently expensive and require specialized facilities and training. The instruments are still at early commercial stages and are not yet universally available beyond research hospitals. In addition, therapists must undergo training not only in operating the equipment but also in interpreting real-time cavitation signals and adjusting treatment parameters accordingly. The steep learning curve may hinder swift incorporation into standard oncology practice [16,40]. Finally, although histotripsy for liver tumor treatment has been approved by the U.S. FDA, approvals in other nations have yet to materialize, and standardized treatment protocols, patient selection guidelines, and reimbursement policy have yet to be defined. Overall histotripsy for liver cancer is presently limited to specialized centers, equipped with ultrasound-guided systems and skilled personnel, and the procedure entails higher upfront costs due to advanced equipment and expertise.

Lastly, existing clinical evidence, while promising, is currently narrow in scope. The majority of human trials thus far have included small groups with comparatively small tumors (usually <3 cm) and limited follow-up intervals. It is unknown how effectively histotripsy is for larger or multifocal tumors, or how it stands against head-to-head comparison with known modalities such as MWA, RFA, or stereotactic body radiotherapy. Multicenter randomized trials with meaningful endpoints in the future are necessary to answer these questions.

## 5. Future Directions and Research Priorities

With histotripsy evolving from an experimental modality to a useful clinical device, there are ample research opportunities to optimize and guide its clinical utility, define its efficacy, and help set its niche among the numerous liver cancer therapeutic modalities

At this point, the most anticipated focus of research is a multi-center randomized controlled trial. While preliminary human trials have reported promising technical success and safety, the small numbers of patients and short follow-up times preclude conclusions regarding long-term oncologic results. Future trials will be needed to compare histotripsy to established treatments such as RFA, MWA, and surgical resection in HCC and colorectal liver metastases. End points would include local control rates, recurrence-free survival, overall survival, quality of life, and cost-effectiveness.

A second critical focus for research is the integration of histotripsy with systemic therapy, e.g., immunotherapy. Histotripsy has already been shown in preclinical models to release intact tumor antigens and DAMPs during ablation of the tumor, which may stimulate the immune system and enhance the effectiveness of immune checkpoint inhibitors. Combination regimens will offer new options for advanced or multifocal disease, particularly in the surgery contraindicated patient.

Technological advancement remains the foundation upon which future progress is built. Current histotripsy machines are large, expensive, and suitable for lesions with good acoustic windows. Further developments to smaller, less expensive machines with improved imaging integration and beam steering will expand the clinical applications of histotripsy. New transducer technologies such as phased-array systems with real-time aberration correction might allow more effective energy delivery through thick anatomy, enabling treatment of tumors behind ribs or buried deep in the liver. Moreover, advances in cavitation mapping, artificial intelligence-driven targeting, and robotics guidance can further improve reproducibility and accuracy.

In order to refine clinical applications, research is also needed to establish standardized protocols for patient selection, treatment planning, and follow-up. Whether the optimal number of pulses is known, the treatment volume margins, the repeatability criteria, or the optimal imaging modalities for intra-procedure guidance and post-procedure assessment are not yet established. Setting biomarkers for response prediction to histotripsy, such as tumor stiffness, vascularity, or molecular fingerprint, would enable individualized treatment planning.

Finally, uses of histotripsy may be found beyond curative ablation. Investigating the role of histotripsy as a neoadjuvant or bridging therapy, e.g., prior to liver transplant or systemic therapy, adds further research strategies in the treatment of unresectable or borderline resectable patients. Moreover, its ability to debulk lesions without sacrificing critical vasculature or ducts will make it an option for palliation in selecting biliary obstruction or bulky symptomatic neoplasms. This broadens the application of histotripsy from ablation to debulking.

Histotripsy has substantial promise in the management of malignancies and benign diseases beyond liver tumors, including for renal tumors, pancreatic tumors, and benign prostatic hyperplasia (BPH) [32,40]. In renal tumors, active early-phase clinical trials and preclinical work demonstrate the capability to produce well-defined, accurate ablation zones with sparing of vital structures and limiting collateral damage [55]. For pancreatic tumors, histotripsy has proven safe and effective in animal models, with possible therapy for lesions near sensitive anatomical structures that preclude thermal ablation [56]. For BPH, clinical trials have determined effective debulking of tissue with a safety profile analogous to liver applications. Twenty-five men were treated with histotripsy for BPH in a report by Schuchter et al. [57], and no intraoperative adverse events were observed; postoperative minor device-related effects were transient urinary retention, microscopic hematuria, and one severe urinary retention, while drastic improvement in the International Prostate Symptom Score was observed at 1, 3, and 6 months, irrespective of limited imaging evidence of prostate tissue debulking. In all these studies, outcomes are generally consistent with outcomes in liver tumors, with high technical success and precise ablation, and minimal serious complications.

## 6. Conclusions

Histotripsy offers a groundbreaking, non-invasive approach to liver tumor ablation, combining precision, safety, and the potential for immune activation. Early clinical and preclinical data support its effectiveness, particularly in challenging cases near vital structures. While further trials are needed to confirm long-term outcomes and broader applicability, histotripsy holds strong promise as a next-generation modality in liver oncology.

## Figures and Tables

**Table 1:** Summary of Clinical Trials Using Histotripsy for Liver Tumors. Abbreviations: HCC, hepatocellular carcinoma; CRLM, colorectal liver metastases.

Study Name	Phase	*n* Patients	Tumor Types	Technical Success	Major Complications
Vidal-Jove et al., 2022 [34]	I	8	HCC, metastases	100%	0%
Wah et al., 2023 [35]	I/II	44 tumors	HCC, CRLM, others	95%	7%
Wehrle et al., 2025 [36]	—	230	Broad spectrum	High (implied)	1.3%

**Table 2:** Contraindications and limitations of histotripsy. Abbreviations: GI, gastrointestinal.

Category	Contraindications
Imaging Limitations	Deep tumors, overlying gas, obesity
Coagulopathy	Severe thrombocytopenia, uncorrectable coagulopathy
Tumor Type	Diffuse/infiltrative tumors, extensive liver burden
Liver Function	Advanced dysfunction (e.g., Child–Pugh class C)
Anatomical Risk	Tumors near GI tract, gallbladder

## Data Availability

No new data are created.
